# Surgery

**Published:** 1908-03

**Authors:** E. W. Hey Groves


### SURGERY.

A very great change came over the methods and results of surgical operations when accurate ligatures were substituted for avulsion and the actual cautery in the treatment of wounded vessels. A further great advance has now been made in the **surgery of the blood-vessels,** for it has been shown that their walls can be submitted to suture without occlusion of their lumen, and thus wounds in their walls may be sutured, venous, arterial or arterio-venous anastomosis may be deliberately undertaken, large vessels opened and subjected to a search for emboli or submitted to plastic operations. Pearce Gould,[1] (just) after Ballance and Edmunds'[2] important work had appeared on the ligature of arteries in their continuity, says "the only reliable treatment of a punctured wound of an artery is the aseptic ligature of the vessel above and below the puncture." But the recent work has abundantly demonstrated that this position may now be abandoned, and that the walls of large arteries may and ought to be sutured in such a way as to leave their lumen free. It will be convenient to describe the subject under the headings of (1) Arterial suture, (2) Vascular anastomosis, (3) Arteriotomy for embolus, and (4) the plastic operations for aneurism, rather than to attempt any historical account of the various steps in the progress of this important change.

**Arterial suture.**—According to American authors, it was an English surgeon, Hallowel,[3] who first, in 1759, conceived the idea of treating an arterial wound without bringing about occlusion of its lumen. His patient had a punctured wound of the brachial artery, and he placed a pin through the edges of the wounded artery, and wound a ligature round its ends. In this case success resulted, but it must have been in spite of all the dangers of the septic era of surgery. It is only within recent years and under the most careful aseptic methods that any considerable series of cases has been presented of successful arterial suture. Jassinowsky,[4] in 1889, proved the feasibility of the method in animals, and he was followed by many other workers, so that in 1902 Schmitz[5] was able to collect twenty-one cases of the suture of lateral wounds of the large arteries (axillary, femoral, popliteal brachial, common iliac, common carotid, internal carotid) in the human subject, and to formulate the conditions necessary for success. These were: Asepsis, the use of very fine round needles and silk, the employment of the continuous suture (which included all the coats of the vessel), and temporary hæmostasis carried out by

[1] *Treves's System of Surgery*, vol. i., 1895, p. 501.
[2] *The Ligature of Arteries in Continuity*, 1891.
[3] Watts, *Ann. Surg.*, 1907, xlvi. 374.
[4] Jassinowsky, *Die Arteriennaht*, Dorpat, 1889.
[5] Quoted by Watts, *loc. cit.*

means which imposed no injury of the vessel wall. And to these may be added that the sheath of the vessel should be separated from it by a clean dissection for as short a distance as is compatible with the arterial suture, and then separately sewn over the wounded vessel as a protection. The whole secret of success lies in the promotion of healing in the walls of the vessels without producing a thrombus. It is well known that the liability to the latter formation depends more upon injury to the intima of the vessel than anything else. Hence it is that no clamps can be used for the vessel wall unless they are carefully guarded by rubber, and have broad smooth surfaces. The edges of the wounded artery must not be touched with forceps, the needles must have no cutting edges, and the silk must be lubricated by sterilised vaseline.

**Vascular anastomosis or the circular suture of vessels.**—The next accomplishment in arterial suture was the circular or end-to-end union of vessels, and, curiously enough, the advocacy of methods for doing this has gone through the same stages as in the case of intestinal anastomosis. That is to say, the first stage consisted in showing that end to end stitching was difficult, and often resulted in failure; then the mechanical contrivance was introduced—a bobbin, in fact—to replace the necessity for stitching; and, lastly, it has been recognised that, after all, accurate stitching without reliance on any mechanical method gives the best results. Murphy,[1] in 1897, began experimental work on the circular suture of arteries, and he introduced the invagination method, by which the proximal end of a divided artery was drawn within the lumen of the distal end by sutures which were tied outside the wall of the latter, a circular suture completing the operation, and in the same year he was able to carry this out successfully in the case of a man whose femoral artery had been severed by a bullet in Scarpa's triangle. In 1900 Payr[2] introduced his ring or anastomosis button, made of magnesium, a substance which he averred was absorbed by the tissues. This has quite recently been improved by Crile,[3] who has added a little handle to it, which makes it much easier of manipulation. It consists of a small tube, about $\frac{1}{4}$ inch long, corresponding to the diameter of the vessel to be sutured. On its outer surface are two grooves running round its circumference. One end of the artery is threaded through the lumen of the tube, and then folded back like a cuff and tied by a thread on the upper groove. It will thus have its intima outwards. Over this the other end of the artery is coaxed and tied round the lower groove, the two vessels having thus their intimal surfaces in apposition. This apparatus has been used with great success by Crile in the immediate transfusion

[1] Quoted by Watts, *loc. cit.*

[2] Payr, *Verhandl. d. deutsch. Gesell. f. Chir.*, 1900, p. 31.

[3] *Ann. Surg.*, 1907, xlvi. 329; *Rev. méd. du Canada*, 1904–5.

of blood, the central end of the radial artery of the donor being joined to the central end of a superficial vein of the recipient. But it remains to be shown whether this method presents any real advantages over saline transfusion. Carrel,[1] in 1902, introduced his method of suture, which in his own hands and in those of his co-workers has given such brilliant results. It consists in first obtaining a clean-cut edge of both arterial ends, then bringing these together by three tension sutures equidistant from one another, and finally sewing the edges by a continuous fine silk suture without everting them at all. The very simple and yet important point consists in the value of the tension sutures in bringing the edges of the vessel into accurate apposition, as the sutures are held up two at a time. By this method not only have severed arteries been united, but arteries have been united to veins, sections of veins have been substituted for arteries, and whole organs with their arteries and veins have been transplanted from one animal to another. At present this work is chiefly experimental, for although several times it has been attempted in man to substitute the saphenous vein for a blocked femoral artery, none of them have been permanently successful.

Nevertheless, the scientific interest of this work is very great, and will certainly lead to valuable clinical success in the future. As a wonderful example of what may be achieved in this direction, the following may be selected. Carrel and Guthrie[2] removed both kidneys and ureters, together with a portion of the aorta and vena cava, from a dog, and transplanted them into the abdomen of another dog, implanting the great vessels into those of the new host after a corresponding portion of his own organs had been removed. The dog lived for eight days, and secreted copious urine. Watts gives an account of forty-three experiments on the great vessels in the neck and groin of dogs, and his beautiful illustrations, taken at periods varying from three weeks to three months afterwards, show clearly how accurately the wounded vessels will unite with practically no diminution of their calibre. These include cases of end-to-end junction of divided arteries, one artery joined to another, arteries joined to veins, and veins substituted for arteries.

**Arteriotomy for embolus and thrombosis.**—It is clear that if wounds of arteries can be successfully sutured, then an artery may be opened with impunity in order to remove a thrombus or embolus; and this has been repeatedly proved in clinical cases—that is to say, large arteries, *e.g.* the external iliac, femoral and popliteal have been opened, their contents removed, and then they have been sewn up without the occurrence of any recurrent or secondary hemorrhage. But it must be reluctantly admitted that so far in none of these cases has complete success

[1] Carrel, *Lyon méd.*, 1902, xcviii., 859.

[2] Carrel and Guthrie, *Science*, N.Y., 1905; *Am. Med.*, 1905, x. 284.

been achieved, for although gangrene seemed to be averted or delayed for a time, yet it always occurred later and required amputation. Sabanajew,[1] in 1896, thus opened a femoral artery for a rheumatic embolus, which he failed to find. Lejars,[2] in 1902, removed a thrombus from the external iliac artery, but subsequent thrombosis occurred, and necessitated amputation a month later. Stewart,[3] in 1905, in a case of threatened gangrene after contusion of the inguinal region, opened the popliteal artery, and finding nothing, having closed this, opened the femoral artery and removed a displaced atheromatous patch. He excised the diseased part of the artery and sewed the two ends together; but the gangrene was not arrested, and amputation had to follow. The same surgeon removed an embolus from the bifurcation of the femoral artery, but after a temporary promise of success the leg had to be removed below the knee six weeks later. Sampson Handley[4] has recently related a very similar case.

**The plastic operations for aneurism.**—It is now more than ten years since Matas[5] invented his new operative treatment of external aneurisms, and although it has been followed by very successful results, it is remarkable how little attention or even notice has been bestowed upon it in English text-books. It consists essentially in opening the sac of the aneurism and dealing with it from within. The latter procedure may take three different forms. (1) The obliterative operation consists in finding all the vessels which open into the sac, and sewing together their open mouths by silk stitches. The sac wall is then plicated, and sewn as a sort of pad beneath the skin. According to Gibbon,[6] this operation has been carried out twenty-four times, and in not one case has relapse or recurrent hemorrhage occurred, but in one (Frazier[7]) it led to gangrene. The latter case was an aneurism which involved the common femoral at its bifurcation, and its treatment involved obliteration of both superficial and deep vessels. (2) The restorative operation is only concerned with a distinctly saccular aneurism with a narrow neck of communication with the parent artery. This opening is sewn together with interrupted silk sutures, and the lumen of the parent trunk is left unobliterated. The ideal conditions which allow of its execution are, of course, somewhat rare. It has been performed eight times, most of the cases dealing with popliteal aneurisms. In all the result has been successful, and no relapses have occurred. (3) The reconstructive operation. In this case a more or less fusiform aneurism is dealt with, or, at any rate,

[1] Quoted by Stewart, *Ann. Surg.*, 1907, xlvi., 342.
[2] *Bull. et Mem. Soc. de Chir. de Paris*, 1902, xxviii.
[3] *Ann. Surg.*, 1905, xlii. 621. [4] *Brit. M. J.*, 1907, ii. 712.
[5] *Med. News*, 1888, liii. 462; *Ann. Surg.*, 1903, xxxvii. 161.
[6] *Ann. Surg.*, 1907, xlvi. 366. [7] *Ibid.*, p. 358.

one whose cavity is not separable from that of the parent trunk. After opening the sac, a rubber tube is laid along its floor, running from the afferent to the efferent trunk. Over this the walls of the sac are sewn by interrupted Lembert sutures, the tube being removed before the last stitches are tied. This has been carried out in seven cases, but in two of them relapse, *i.e.* re-formation of the aneurism has occurred. At first sight it may appear as if the obliterative operation were only a return to the old operation of Antyllus, whereby the sac was first incised and then excised with ligature of all bleeding points. But the cardinal distinction consists in the fact that the outside of the sac is not touched except in opening it, and thus the veins and collateral arteries, which are so often closely associated with the sac wall, escape the risk of injury.

E. W. Hey Groves.

